# Insulin resistance, Ca^2+^ signaling alterations and vascular dysfunction in prediabetes and metabolic syndrome

**DOI:** 10.3389/fphys.2025.1535153

**Published:** 2025-06-10

**Authors:** Tatiana Romero-García, J. Gustavo Vázquez-Jiménez, Rommel Sánchez-Hernández, J. Alberto Olivares-Reyes, Angélica Rueda

**Affiliations:** ^1^ Department of Biochemistry, Center for Research and Advanced Studies (Cinvestav) of the National Polytechnic Institute, México City, Mexico; ^2^ Facultad de Medicina, Universidad Autónoma de Baja California, Mexicali, Mexico; ^3^ Department of Physiology, Biophysics and Neurosciences, Center for Research and Advanced Studies (Cinvestav) of the National Polytechnic Institute, México City, Mexico

**Keywords:** prediabetes, metabolic syndrome, vascular dysfunction, insulin signaling, calcium signaling, SERCA pump, calcium sparks, ryanodine receptors

## Abstract

Prediabetes and Metabolic Syndrome (MetS) share a common pathway to induce vascular dysfunction through hyperinsulinemia without the presence of overt hyperglycemia. Insulin resistance (IR) is a key factor in vascular complications in diabetes; however, vascular dysfunction has been reported in MetS patients, even in the absence of chronic hyperglycemic conditions. We consider that the alterations in the intracellular Ca^2+^ handling of vascular smooth muscle cells (VSMCs) and the impairment of the insulin receptor signaling pathway may contribute to the etiology of vascular diseases in prediabetes and MetS. Therefore, it is critical to understand the mechanisms by which prediabetes and MetS alter the expression and activity of proteins involved in intracellular Ca^2+^ signaling in VSMCs, particularly those related to vasorelaxation. The functional unit, integrated by the voltage-gated L-type Ca^2+^ channel (Ca_V_1.2), the Sarco/Endoplasmic Reticulum Ca^2+^ ATPase (SERCA pump), the ryanodine receptor (RyR), and the large-conductance Ca^2+^-activated K^+^ channel (BK_Ca_), regulates the vascular tone and promotes vasorelaxation of the resistance arteries. Changes in this functional unit may contribute to vascular dysfunction. This review summarizes the most recent knowledge regarding alterations in the expression or activity of these proteins in the vasculature of experimental models with characteristics of prediabetes and MetS.

## 1 Introduction

Vascular diseases have been associated with a high risk of mortality in people diagnosed with Metabolic Syndrome (MetS) (Wassink et al., 2007). MetS factors, such as abdominal obesity, insulin resistance (IR), dyslipidemia, and arterial hypertension, can directly alter the blood vessel function and particularly influence the activity of vascular smooth muscle cells (VSMCs), key structural and functional components of the vasculature (Aoqui et al., 2014). The middle layer (or *tunica media*) of arterial blood vessels contains VSMCs between the endothelial cells (ECs) and the more external layer of the blood vessels (or *tunica adventitia*). The VSMCs embedded in resistance-sized arteries (<400 μm in lumen diameter) play a key role in maintaining the vascular tone and regulating the myogenic response because of their contraction and relaxation capabilities (Aoqui et al., 2014; Jackson, 2020). A vast diversity of intracellular Ca^2+^ signals control excitation–contraction and relaxation mechanisms in these cells. The global increment of cytoplasmic Ca^2+^ concentration ([Ca^2+^]_cyt_) induces VSMC contraction. Once Ca^2+^ reaches the deep cytoplasm of VSMCs via the opening of diverse plasma membrane channels such as the voltage-gated L-type Ca^2+^ channel subunit α (Ca_V_1.2), it binds to calmodulin (CaM). The Ca-CaM complex activates the myosin light chain kinase (MLCK). This allows active myosin cross-bridges to slide along actin filaments, creating muscle tension. The Sarco/Endoplasmic Reticulum Ca^2+^ (SERCA) pump recaptures the cytoplasmic Ca^2+^ into the Sarcoplasmic Reticulum (SR), increasing the SR Ca^2+^ load. The luminal Ca^2+^ promotes the activation of clusters of Ryanodine Receptors (RyRs), which release Ca^2+^ as Ca^2+^ sparks. These elementary, local Ca^2+^ events activate the nearby large-conductance Ca^2+^-activated K^+^ channels (BK_Ca_) that generate spontaneous transient outward currents (STOCs), which induce hyperpolarization and lead to vasorelaxation (Nelson et al., 1995; Essin and Gollasch, 2009; Fernández-Velasco et al., 2014). Therefore, the tetrad formed by Ca_V_1.2, SERCA pump, RyRs, and BK_Ca_ channels constitutes a functional unit that regulates the vascular tone and counteracts vasoconstriction. Changes in the activity or expression of any of these proteins can impact vascular function. This review focuses on the vascular dysfunction in prediabetes and MetS and summarizes the most recent knowledge regarding alterations in the expression or activity of the proteins that form the functional unit (Ca_V_1.2, SERCA pump, RyRs, and BK_Ca_ channels) that participates in regulating the vascular tone, in experimental models with characteristics of prediabetes and MetS.

### 1.1 Definitions of prediabetes

The term prediabetes was coined in the 1970s and describes individuals whose fasting glycemia does not meet the criteria for type 2 Diabetes Mellitus (DM2) diagnosis but is high enough (≥100 and ≤125 mg/dL) to be considered as normal (American Diabetes Association, 2021; Gaitán-González et al., 2021). Prediabetes constitutes a reliable indicator of high risk to develop DM2 and macrovascular disease (Rett and Gottwald-Hostalek, 2019); however, in the clinical environment, its diagnosis has been complicated because of the lack of performing the oral glucose tolerance test on a regular basis. The National Diabetes Data Group defined prediabetes as a condition characterized by impaired glucose tolerance (IGT) after 2 h of post oral glucose tolerance test (with blood glucose values between 140 and 199 mg/dL) (American Diabetes Association, 2021).

By the end of the 1990s, the American Diabetes Association and the World Health Organization (WHO) included impaired fasting glycemia (IFG) by showing fasting blood glucose levels (in mg/dL) from 100 to 125 or from 110 to 125, respectively, as a key parameter for prediabetes diagnosis. Finally, the American Diabetes Association incorporated the glycated hemoglobin A1c (HbA1c) criterion for the prediabetes definition. Currently, prediabetes is defined as IFG with blood glucose levels between 100 and 125 mg/dL, the presence of IGT, and/or HbA1c in a range of 5.7%–6.4% (American Diabetes Association, 2021). Nowadays, prediabetes is also associated with obesity, high levels of total cholesterol and/or triglycerides, and low levels of HDL-cholesterol (HDL-C), which are also common alterations in MetS (Punthakee et al., 2018).

### 1.2 Definitions of metabolic syndrome

MetS is defined as an association of diverse physiological and biochemical disturbances that constitute a major risk factor for the development of DM2 and cardiovascular disease (CVD) (Alberti et al., 2009). MetS is also associated with a wide range of pathologies like non-alcoholic fatty liver disease, polycystic ovary syndrome, obstructive sleep apnea, sexual dysfunction, and even breast, colon, and prostate cancers (Gaitán-González et al., 2021). The incidence of MetS has been rising concomitantly with insufficient physical exercise and unhealthy dietary habits in both young and adult populations worldwide. MetS prevalence depends on diverse factors such as age, gender, and ethnicity, with different studies indicating that about a quarter of the adult worldwide population has been diagnosed with MetS, and its incidence emulates the obesity and DM2 rates (Gupta and Gupta, 2010; Saklayen, 2018). For instance, in Mexico, the prevalence of MetS in individuals without incident DM2 is 43.9%, and abdominal obesity is 78.1% (Arellano-Campos et al., 2019). However, there is a three-fold higher risk of developing DM2 in subjects who had MetS compared to those who did not (Arellano-Campos et al., 2019). Therefore, there is an urgent need to control and prevent prediabetes and MetS to diminish the occurrence of DM2 and its adverse consequences on the cardiovascular system.

While the existence of MetS is not in doubt, a worldwide agreement concerning the main corporal and biochemical parameters for MetS diagnosis has been hard to achieve. Over the past years, several clinical organizations have tried to establish the criteria to best describe the signs and symptoms that characterize the metabolic risk of MetS. Different organizations, such as the WHO, the Joint Interim Statement by the International Diabetes Federation, the National Heart, Lung, and Blood Institute (NHLBI), the American Heart Association (AHA), the World Heart Federation, the International Atherosclerosis Society, and the International Association for the Study of Obesity have established a definition as well as a standard for MetS diagnosis (Table 1). The harmonized definition of MetS establishes that a person must fulfill at least three of the following criteria: central obesity, hypertriglyceridemia, high blood pressure, impaired fasting glycemia (a common feature with prediabetes), and low levels of HDL-C to be diagnosed with MetS (Alberti et al., 2009; Neeland et al., 2024). It is also important to prevent the progression of MetS to more advanced stages, including end-organ damage such as the kidney, to avoid the emergence of the cardiovascular-kidney-metabolic syndrome (Neeland et al., 2024).

**TABLE 1:** Different criteria for Metabolic Syndrome diagnosis.

Feature	Organization, year						
	WHO, 1999 (Alberti and Zimmet, 1998)	EGIR, 1999 (Balkau and Charles, 1999)	NCEP-ATP III, 2001 (Cleeman, 2001)	AACE, 2003 (Einhorn, 2003)	AHA/NHLBI, 2005 (Eckel et al., 2005; Grundy, 2006)	IDF, 2006 (Alberti et al., 2006)	JIS, 2009 (Alberti et al., 2009)
Obesity	Waist/hip ratio >0.9 (men) or >0.85 (women) or BMI >30 kg/m2	Waist circumference >94 cm (men) or >80 cm (women)	Waist circumference >102 cm (men) or >88 cm (women)	Not considered	Waist circumference >102 cm (men) or >88 cm (women)	Waist circumference >94 cm (men) or >80 cm (women)	Elevated waist circumference (According to country and population-specific conditions)
Blood Pressure (mmHg)	>140/90	>140/90 or hypertension drug treatment	>130/85 or hypertension drug treatment	>130/85 or hypertension drug treatment	>130 systolic blood pressure or >85 diastolic blood pressure or hypertension drug treatment	>130/85 or hypertension drug treatment	>130/85 or hypertension drug treatment
Glycemia (mg/dL)	>110 or IR (HOMA-IR)	>110 without diabetes diagnosis	>110 or elevated glucose drug treatment	Fasting values between 110 and 125 or impaired glucose tolerance	>110 or elevated glucose drug treatment	>100 or diagnosed diabetes	>100 or elevated glucose drug treatment
Triglycerides (mg/dL)	>150	>175 or dyslipidemia treatment	>150 or elevated triglycerides drug treatment	>150	>150 or elevated triglycerides drug treatment	>150 or elevated triglycerides drug treatment	>150 or elevated triglycerides drug treatment
HDL-C (mg/dL)	<35 for men or <40 for women	>40 or dyslipidemia treatment	<40 for men or <50 for women or drug treatment for low HDL-C	<40 for men or <50 for women	<40 for men or <50 for women or drug treatment for low HDL-C	<40 for men or <50 for women or drug treatment for low HDL-C	<40 for men or <50 for women or drug treatment for low HDL-C
Other	Albumin excretion >20 μg/min	IR or fasting hyperinsulinemia (HOMA-IR)					
Diagnosis	IGT, or elevated glycemia plus two or more parameters	IR or fasting hyperinsulinemia plus two or more parameters	The presence of three or more parameters	High risk of IR along with two or more parameters presence	The presence of three or more parameters	Elevated waist circumference along with two or more parameters presence	The presence of three or more parameters

AACE, American Association of Clinical Endocrinology; AHA/NHLBI, American Heart Association/National Heart, Lung and Blood Institute; BMI, Body mass index; EGIR, European Group for the study of Insulin Resistance; HDL-C, High density lipoprotein cholesterol; HOMA-IR, Homeostatic Model Assessment for Insulin Resistance; IDF, International Diabetes Federation; IGT, Impaired glucose tolerance; IR, insulin resistance; JIS, Joint Interim Statement; NCEP-ATP III, National Cholesterol Education Program–Adult Treatment Panel III; WHO, World Health Organization.

Due to its high probability of leading to the development of DM2, it has long been debated whether MetS should be considered as a prediabetic state or not, independently of the presence of IFG. For instance, MetS is not necessarily a prediabetic state because it may occur without the presence of IR (Meigs et al., 2007). This notion is associated with the AHA/NHLBI definition for the MetS, which was structured to simplify the criteria for MetS to standard blood test parameters for facilitating its diagnosis and its focus on obesity as a requirement (Grundy, 2005).

**FIGURE 1:**
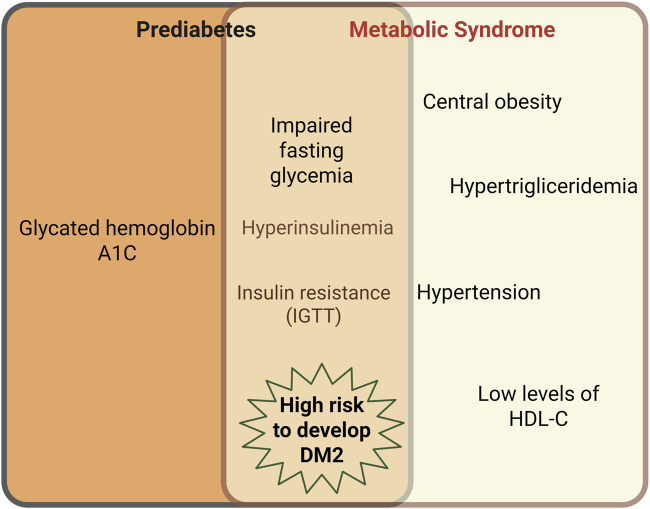
Intersection between prediabetes and MetS characteristics. Although prediabetes and MetS are different clinical entities, both share the impaired fasting glycemia (IFG) in their criteria for diagnosis. IFG underlies hyperinsulinemia and insulin resistance, the latter, indirectly determined by the impaired glucose tolerance test (IGTT). Both MetS and prediabetes can trigger type 2 Diabetes Mellitus (DM2). Created in BioRender.

On the other hand, an insulin blood test is not a frequently requested exam by clinicians for prediabetes diagnosis, and it does not lend itself as well to large epidemiological studies, where a quick and simple insulin assessment is important (Huang, 2009). Several experts have considered that MetS definitions that exclude hyperinsulinemia or IR as part of the MetS components fail to provide a proper MetS diagnosis (Reaven, 2005). In fact, when the euglycemic clamp test is carried out in people with high visceral fat, there exists a close relationship with the presence of IR (Ruderman et al., 1998). IR is an entity where the insulin signaling pathway is altered at several signal transduction points and favors the development of dyslipidemia, obesity, and arterial hypertension (Ruderman et al., 1998; Eckel et al., 2005); then, IR should be considered as part of MetS pathophysiology. Moreover, experimental MetS models develop hyperinsulinemia and IR in organs with a critical metabolic role, such as the liver and the heart (Özcan et al., 2004; Landa-Galvan et al., 2020). Although prediabetes and MetS are different clinical entities, both share IFG in their criteria for diagnosis. IFG underlies hyperinsulinemia and IR, indirectly determined by the IGT test. Also, both MetS and prediabetes can trigger the development of DM2 (Reaven, 1988; Salazar et al., 2017) (Figure 1).

### 1.3 Vasculopathy in prediabetes and molecular mechanisms

MetS and prediabetes are involved in several microvascular and macrovascular diseases (Palladino et al., 2020). For instance, the Atherosclerosis Risk in Communities study determined that prediabetic patients were 30% more prone to be hospitalized (Schneider et al., 2016). An increase of 1% in the HbA1c was related to an augmented risk in coronary heart disease and 10-year cardiovascular mortality (Khaw et al., 2004; Wasserman et al., 2018). Likewise, the three fundamental microvascular complications: retinopathy, nephropathy, and neuropathy, have been associated with prediabetes in several studies (Brannick et al., 2016).

In patients with IFG, the *in vivo* vasodilatory response to intra-arterial infusion of ACh, an endothelium-dependent vasodilator, was reduced compared to healthy subjects. In contrast, the vasodilatory response to sodium nitroprusside (SNP), which is endothelium-independent, was similar between the two groups, indicating that mainly endothelium-dependent vasodilation was impaired in individuals with prediabetes (Vehkavaara et al., 1999). Another study evaluated both endothelium-dependent and -independent vasodilatory responses with the same vasodilators, reporting impairment of both mechanisms compared to healthy subjects (Caballero et al., 1999). These findings suggest that vascular dysfunction in prediabetes can be heterogeneous, with some individuals exhibiting isolated endothelial impairment, while others present vascular abnormalities that affect both ECs and VSMCs function.

Some studies have suggested that the mechanisms underlying vascular dysfunction in prediabetes may differ from diabetes-associated vasculopathy. Instead of the advanced vascular damage induced by chronic hyperglycemia in DM2, IR in prediabetes may contribute to endothelial and VSMCs dysfunction (Jiang et al., 1999b). In the aorta and microvessels of obese Zucker rats (OZR), insulin signaling is impaired, as evidenced by a reduced tyrosine phosphorylation of the insulin receptor substrates IRS-1 and IRS-2, decreased phosphatidylinositol 3-kinase (PI3K) activity, and diminished serine phosphorylation of Akt upon insulin stimulation (Jiang et al., 1999a).

Although prediabetes and MetS share a common pathway to induce vascular dysfunction through hyperinsulinemia, some studies indicate that the pathophysiology of prediabetes could be related to early pancreatic β-cells dysfunction. This situation leads to a progressive decline in insulin secretion, with no IR establishment, unlike the pathophysiology of DM2 (Abdul-Ghani and DeFronzo, 2009; Kanat et al., 2012; Kabadi, 2017). Therefore, hyperinsulinemia is not always a factor participating in the vascular dysfunction in prediabetes.

### 1.4 Vasculopathy in MetS and molecular mechanisms

MetS correlates well with the development of several cardiac diseases, including diastolic dysfunction, heart attacks, and arrhythmia (Dincer, 2012; Tune et al., 2017), underlying macrovascular and microvascular diseases. Several studies have shown that MetS is a risk factor for coronary artery (CA) disease (Carr and Brunzell, 2004) and arterial thickness and stiffness, which lead to disturbances in blood pressure (Scuteri et al., 2004; Czernichow et al., 2005). Also, MetS impairs microvascular peripheral cerebral perfusion (Nazzaro et al., 2013) and increases stroke events (Ford, 2004).

Although hyperglycemia is a crucial factor of vascular complications in diabetes, vascular dysfunction has also been reported in MetS patients even in the absence of an overt hyperglycemic condition (Galassi et al., 2006; Rundek et al., 2010); therefore, more attention is needed to understand the effects of dyslipidemia, hyperinsulinemia, and IR as key factors of vascular complications.

While in prediabetes endothelial dysfunction is a precursor to more severe vascular issues, in MetS the inflammation and the oxidative stress are more pronounced, particularly involving tumor necrosis factor-α (TNF-α). Small arteries of OZR have impaired endothelium-dependent vasodilation in response to ACh, whereas endothelium-independent vasodilation in response to SNP was comparable with control animals, along with decreased endothelial nitric oxide synthase (eNOS) expression, increased both mRNA and protein expression of TNF-α, and reactive oxygen species (ROS) (Picchi et al., 2006). Increased levels of TNF-α have also been found in the obese (*ob/ob*) mouse model (Hotamisligil et al., 1993).

In vascular tissue, ROS are generated in both ECs and VSMCs, with their production being exacerbated in MetS (López-Acosta et al., 2023). For instance, VSMCs or ECs isolated from the aorta and cultured with a high concentration of the free fatty acid palmitate (200 µM), mimicking a dyslipidemic environment, exhibited increased ROS production, which was associated with augmented diacylglycerol synthesis and PKC activity (Inoguchi et al., 2000). Elevated ROS levels promote the transition of VSMCs from a contractile to a proliferative phenotype (Badran et al., 2020), contributing to increased *tunica media* thickness and impaired vasodilatory response.

The etiology of vasculopathy in prediabetes and MetS may share common mechanisms involving alterations in the insulin receptor (InsR) signaling pathway, which affect both ECs and VSMCs. However, specific vascular alterations in MetS, such as increased ROS production and a proinflammatory state, may contribute to distinct pathological features.

Nevertheless, we propose that alterations in intracellular Ca^2+^ handling, as well as changes in the expression or activity of the four proteins involved in regulating the vascular tone (the Ca_V_1.2, the SERCA pump, the RyRs, and the BK_Ca_ channels) in VSMCs impair the vascular function. These mechanisms will be further discussed in the following sections.

## 2 Insulin signaling in the vasculature

### 2.1 Insulin signaling

Insulin is a 51-amino acid peptide hormone secreted by pancreatic β-cells, which plays a fundamental role in maintaining energy homeostasis by regulating glucose and lipid metabolism. To accomplish this, insulin triggers glucose uptake in the muscle and adipose tissues and promotes glycogenesis in the liver and muscle. Insulin also suppresses glyconeogenesis in the liver and exerts a significant effect on lipid metabolism, including fatty acid and triglyceride synthesis and inhibition of lipolysis (Gutiérrez-Rodelo et al., 2017). Insulin elicits critical biological effects in cardiovascular tissues, including VSMCs, such as vasorelaxation, by stimulating nitric oxide (NO) production, decreasing [Ca^2+^]_cyt_, and enhancing myosin light chain (MLC) sensitization through autocrine and paracrine mechanisms (Sowers, 2004).

### 2.2 Metabolic actions of insulin

**FIGURE 2:**
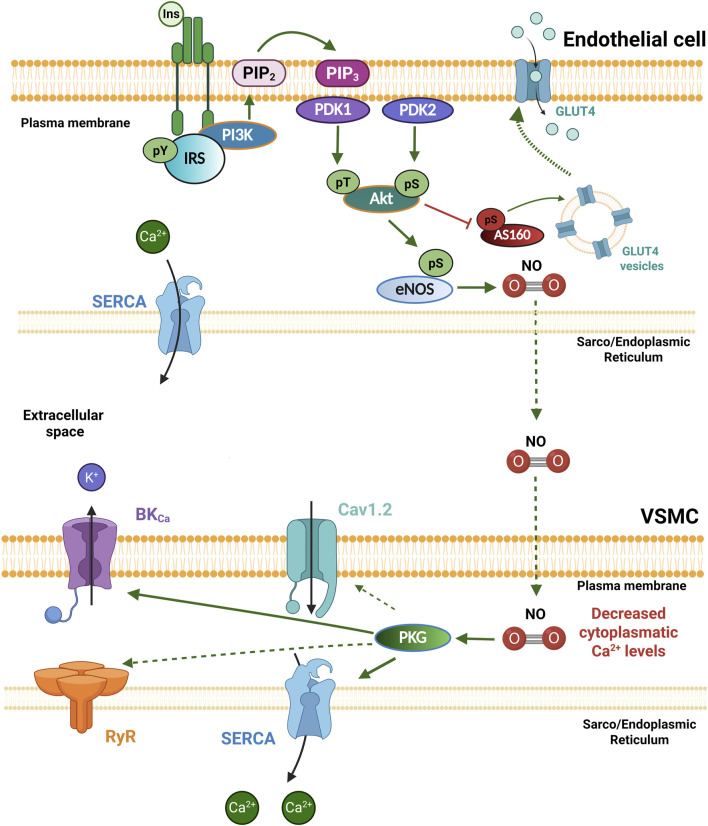
Insulin receptor signaling in the vasculature. In physiological conditions, Insulin (Ins) binds to the insulin receptor (InsR) and promotes its own phosphorylation and the phosphorylation of the IRS on tyrosine residues (pY) as part of the PI3K/Akt signaling cascade, where Akt can be activated via its phosphorylation by phosphoinositide-dependent protein kinases PDK1 and PDK2. Akt regulates glucose uptake through the inhibitory phosphorylation of AS160, increasing the trafficking of GLUT4 storage vesicles to the cell membrane, allowing glucose uptake; and the activation of eNOS, which generates nitric oxide (NO). NO diffuses into the vascular smooth muscle cell (VSMCs) and promotes protein kinase G (PKG) activation, which increases the activity of the SERCA pump and the BK_Ca_ channels, decreasing intracellular Ca^2+^ levels, and promoting vasodilation. Akt, Protein kinase B; AS160, Akt substrate of 160 kDa; BK_Ca_, large conductance Ca^2+^ activated K^+^ channels; eNOS, endothelial nitric oxide synthase; Ins: Insulin; IRS: Insulin receptor substrate; GLUT4, glucose transporter 4; PDK1/2, Phosphoinositide-dependent protein kinase-1/2; PI3K, Phosphatidylinositol-3-kinase; PKG, protein kinase G; PIP2, Phosphatidylinositol bisphosphate; PIP3, Phosphatidylinositol trisphosphate; SERCA, sarco/endoplasmic reticulum Ca^2+^-ATPase. Created in BioRender.

Insulin plays a critical role in the cardiovascular system, where it regulates cardiac contractility, vascular tone, lipid, glucose, and protein metabolism (Muniyappa et al., 2007; Landa-Galvan et al., 2020). The biological actions of this hormone begin when it binds to the InsR, a tetrameric membrane protein with intrinsic tyrosine kinase activity, which undergoes auto-phosphorylation and promotes the phosphorylation of several intracellular scaffolding substrates, such as the IRS on tyrosine residues (pY). This substrate subsequently functions as a docking protein for downstream signaling molecules, activating different signaling pathways (Saltiel, 2021). The primary signaling pathways activated in response to insulin are: 1) the phosphatidylinositol 3-kinase/protein kinase B (PI3K/Akt) pathway, which is responsible for most of their metabolic actions. IRS serves as a scaffold protein for PI3K, which favors the conversion of phosphatidylinositol diphosphate (PIP2) to phosphatidylinositol triphosphate (PIP3), stimulating Akt activation via phosphorylation by the phosphoinositide-dependent protein kinases PDK1 and PDK2; and 2) the mitogen-activated kinase (MAPK) pathway, which regulates gene expression, cellular growth, and proliferation (Olivares-Reyes et al., 2009; Gutiérrez-Rodelo et al., 2017; White and Kahn, 2021). Subsequently, Akt regulates glucose uptake through the inhibitory phosphorylation of Akt substrate of 160 kDa (AS160), increasing the trafficking of glucose transporter 4 (GLUT4) storage vesicles to the cell membrane and allowing glucose uptake. GLUT4 is expressed in VSMCs and mediates both insulin-dependent and non-insulin-dependent glucose uptake (Figure 2) (Banz et al., 1996; Park et al., 2005).

### 2.3 Actions of insulin in both endothelial and vascular smooth muscle cells

InsRs are present in both ECs and VSMCs in blood vessels. Insulin promotes glucose uptake in these cells through a mechanism involving GLUT4 (Banz et al., 1996; Park et al., 2005). In ECs, insulin induces the production of NO through the PI3K/Akt pathway, which activates eNOS (Figure 2). Endothelial-derived NO diffuses into VSMCs to activate the guanylate cyclase (GC) enzyme, which increases cGMP levels to promote vascular relaxation by Ca^2+^-dependent and Ca^2+^-independent mechanisms. Interestingly, insulin treatment does not alter intracellular Ca^2+^ levels in ECs (Hartell et al., 2005), which suggests that insulin-stimulated NO production is Ca^2+^-independent and mediated by Akt activation. Intriguingly, the expression of both eNOS and inducible NOS has also been detected in VSMCs (Trovati et al., 1999; Muniyappa et al., 2007; Salt, 2013). In fact, insulin stimulation of isolated VSMCs increases NO synthesis by both NOS isoforms in a PI3K/Akt-dependent manner, leading to relaxation via activation of protein kinase G (PKG), which suggests that insulin directly regulates vascular tone (Lee and Ragolia, 2006; Muniyappa et al., 2007; Salt, 2013). PKG mediates vasorelaxation mainly by phosphorylating target proteins such as the myosin phosphatase–targeting subunit and the IP_3_ receptor–associated cGMP kinase substrate (Krawutschke et al., 2015). PKG also phosphorylates the BK_Ca_ channel (Alioua et al., 1998; Thorpe et al., 2017; Moraes et al., 2024) and phospholamban, the negative regulator of the SERCA pump, increasing its activity (Sarcevic et al., 1989; Moraes et al., 2024) (Figure 2). However, alterations in these phosphorylation sites in the context of IR have not been shown. Moreover, it has been reported that PKG phosphorylates the cardiac isoform of RyRs (RyR2), thereby modulating its activity via the activation of the PI3K/Akt/NOS signaling pathway (Baine et al., 2020; Gonano et al., 2022); although it is still unknown whether this occurs in the vasculature. Insulin also inhibits activation of the small GTPase RhoA in a NO/cGMP-dependent manner, leading to increased MLC phosphatase activity. Thus, it is possible that these effects, together with the inhibition of Ca^2+^ influx and the increase in Ca^2+^ efflux, underlie the direct effects of insulin on vascular tone (Salt, 2013). These mechanisms increase blood flow and promote the use of glucose in the target tissues (Gutiérrez-Rodelo et al., 2017).

Although evidence suggests that insulin physiologically regulates vascular tone by decreasing [Ca^2+^]_cyt_ in VSMCs, the precise mechanisms by which this hormone exerts its effects remain to be completely elucidated. Pioneering research conducted in the 1990s demonstrated that insulin modulates agonist-induced increases in [Ca^2+^]_cyt_ through Ca_V_1.2 and alters the activity of MLC phosphatases in VSMCs (Muniyappa et al., 2007). Insulin may attenuate VSMC contractile responses by diminishing agonist-mediated increases in [Ca^2+^]_cyt_, partly by decreasing Ca^2+^ influx through both receptor- and voltage-gated channels (Standley et al., 1991). Although the identity of the insulin-regulated channels in VSMCs and the associated mechanisms are not fully elucidated at present, it is highly probable that Cav1.2 is the target of insulin regulation.

### 2.4 Insulin resistance in the vasculature

IR is a systemic disorder in which cells fail to respond to normal levels of circulating insulin. Under this condition, the highly critical metabolic functions of this hormone, mainly in hepatic, muscular, and adipose tissues, such as glucose uptake and synthesis of glycogen, lipids, and proteins, are altered (Olivares-Reyes et al., 2009; Gutiérrez-Rodelo et al., 2017; Vazquez-Jimenez et al., 2021).

IR is considered a condition associated with prediabetes, MetS, and DM2. It has been identified that the most common alterations that give rise to the IR condition are carried out at the level of the InsR itself and the IRS in the effector molecules downstream of the InsR, such as PI3K and Akt, or by changes in the InsR gene expression or proteins that participate in the pathway, such as GLUT4 transporters. It has been shown that the altered expression of GLUT4 transporters can influence vascular reactivity and thus contribute to vascular disease (Atkins et al., 2015).

At the molecular level, the most common alterations in IR are: 1) a decreased number of InsR and its catalytic activity; 2) an increase in InsR and IRS serine/threonine (Ser/Thr) phosphorylation, followed by increased activity of protein tyrosine phosphatase, and 3) decreased PI3K and Akt activity (Gutiérrez-Rodelo et al., 2017).

The detrimental effects of IR involve VSMCs’ proliferation, vasoconstriction, and proinflammatory activity (Ormazabal et al., 2018).

#### 2.4.1 Insulin resistance and vascular dysfunction in MetS

When IR is present, the physiological effects of insulin are diminished, therefore there is an increase in the synthesis and release of this hormone as a compensation mechanism. Prolonged exposure to high insulin levels in the blood impairs the PI3K/Akt/eNOS axis in ECs, reducing vasorelaxation. The impairment of InsR signaling during prolonged hyperinsulinemia also decreases NO production, which contributes to the development of atherosclerosis and hypertension (Madonna and Caterina, 2009; Wasserman et al., 2018).

**FIGURE 3:**
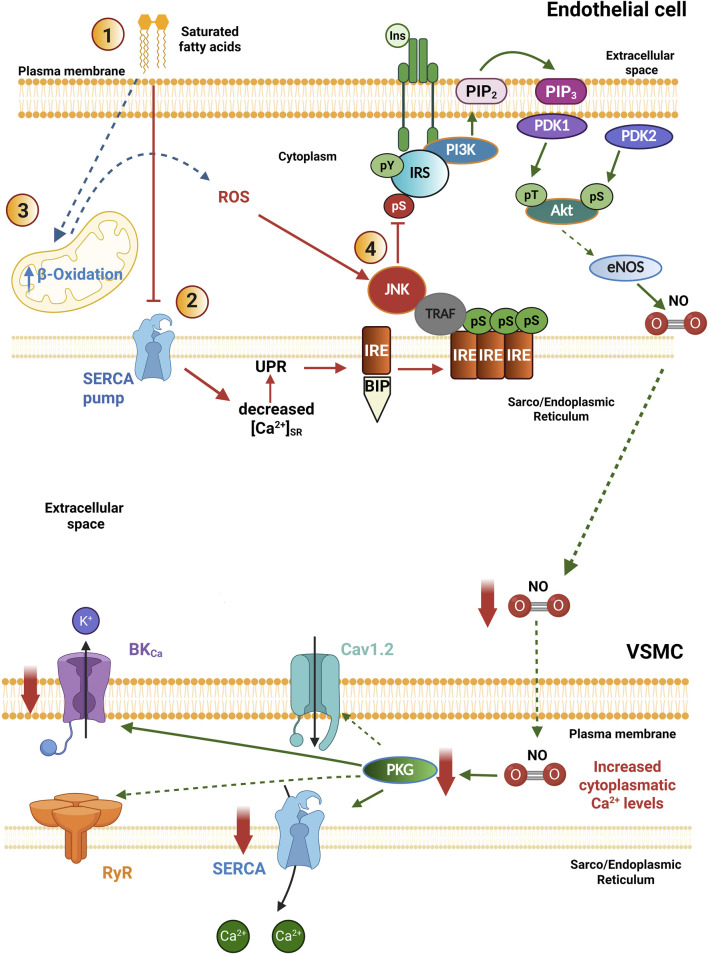
Pathophysiological mechanisms of insulin resistance in the vasculature. In pathological conditions: 1. High concentrations of saturated free fatty acids in the extracellular milieu impair SERCA pump activity, decreasing luminal Ca^2+^ levels in the Sarco/Endoplasmic Reticulum. 2. This condition triggers the unfolded protein response (UPR), involved in the activation of IRE-BIP, whose interaction with TRAF induces JNK activation. 3. In addition, high concentrations of saturated free fatty acids increase mitochondrial β-Oxidation and the generation of ROS, which also favors the activation of JNK. 4. JNK phosphorylates the insulin receptor and the IRS on serine residues (pS), blocking the PI3K/Akt signaling pathway as a mechanism of insulin resistance in blood vessels. The decreased bioavailability of NO levels reduces the activity of SERCA pump and BK_Ca_ channels indirectly by preventing PKG activation, which impairs vasorelaxation. Akt, Protein kinase B; BIP, Binding immunoglobulin protein; BK_Ca_, large conductance Ca^2+^ activated K^+^ channels; eNOS, endothelial nitric oxide synthase; Ins: Insulin; IRS, Insulin receptor substrate; IRE, inositol-requiring endoribonuclease-1; JNK, c-Jun amino-terminal kinase; PDK1/2, Phosphoinositide-dependent protein kinase-1/2; PI3K, Phosphatidylinositol-3-kinase; PKG, protein kinase G; PIP2, Phosphatidylinositol bisphosphate; PIP3, Phosphatidylinositol trisphosphate; ROS, Reactive oxygen species; SERCA, sarco/endoplasmic reticulum Ca^2+^-ATPase; TRAF, TNF receptor-associated factor. Created in BioRender.

Otherwise, high levels of saturated fatty acids can increase the overproduction of ROS in the vasculature, generating oxidative stress and IR (Figure 3) (Inoguchi et al., 2000; Chinen et al., 2007). High ROS levels inhibit insulin-induced Akt activation in vascular cells; the mechanism involved is associated with decreased Tyr IRS-1 phosphorylation. A solid explanation for this mechanism is that high levels of saturated fatty acids stimulate the activation of c-Jun NH2-terminal kinase (JNK) in a process that depends on the increase in ROS production; once activated, JNK increases IRS-1 phosphorylation at Ser residues, inhibiting its activity (Figure 3) (Nakamura et al., 2009). Accordingly, it has been reported that reducing ROS production improves insulin sensitivity in people with diabetes (Brasnyó et al., 2011).

On the other hand, when the hyperglycemic state is not established in MetS, the pathophysiological mechanisms may involve damage to the vasculature, where the endothelium, which is in direct contact with the blood, is the first damaged tissue (Bloom et al., 2023). In this sense, incubations of EC in a culture with high concentrations of saturated fatty acids and the molecular mechanisms involved include the activation of JNK, which impairs InsR signal transduction (Inoguchi et al., 2000; Vazquez-Jimenez et al., 2016; Lopez et al., 2023).

Another hormone that participates in increasing vasoconstriction is aldosterone, which has been found in excess in MetS patients (Briet and Schiffrin, 2011). This mineralocorticoid hormone has a direct effect on the vasculature through activation of the mineralocorticoid receptor (Salazar-Enciso et al., 2018), but also interferes with InsR signaling in both ECs and VSMCs (Bruder-Nascimento et al., 2014). It has been demonstrated that aldosterone impairs InsR signaling by inducing ROS production and altering the PI3K/Akt/eNOS pathway, therefore, mineralocorticoid receptor antagonists have been proposed as therapeutic tools to minimize vascular damage in DM2 (Bruder-Nascimento et al., 2014).

#### 2.4.2 Insulin resistance and ER stress in the vasculature

It has been shown that endoplasmic reticulum (ER) stress is involved in IR at the vasculature (Zhou et al., 2012). Interestingly, obesity and high concentrations of saturated fatty acids in the blood circulation can trigger ER stress, and if this response is not mitigated, IR can evolve into DM2 (Özcan et al., 2004).

ER homeostasis is necessary for the proper maturation of newly synthesized proteins. To maintain homeostasis, high micromolar [Ca^2+^] is required inside this organelle, which is maintained by the SERCA pump (Park et al., 2010). Early events of IR involve high concentrations of saturated fatty acids (for instance, palmitic acid), which promote a decrease in the SERCA pump expression, together with the establishment of ER stress (Vazquez-Jimenez et al., 2016; Galindo-Hernandez et al., 2021). This condition induces the activation of a compensatory mechanism within the ER called the unfolded protein response (UPR), which attempts to restore proper ER activity (Figure 3) (Özcan et al., 2004; 2006; Vazquez-Jimenez et al., 2016).

The UPR triggers the activation of three stress-sensing proteins: the protein kinase R-like endoplasmic reticulum kinase (PERK), the enzyme inositol-requiring endoribonuclease-1 (IRE), and the precursor of transcription factor 6 (ATF6) (Marciniak and Ron, 2006). Physiologically, these three proteins interact through their luminal domain with the chaperone protein BIP/GPR78 (binding immunoglobulin protein/glucose-regulated protein 78), which keeps them inactive (Salvadó et al., 2015). Under low Ca^2+^ conditions in the SR lumen, the activity of chaperone proteins decreases, being unable to fold proteins; this promotes BIP/GPR78 to separate from the sensor proteins to support the correct protein folding. Loss of interaction with BIP/GPR78 induces the activation of sensor proteins, prompting the expression of proteins responsible for protein overload relief (Salvadó et al., 2015; Marciniak et al., 2022). Once IRE-1 is activated, it interacts with the TNF-associated factor favoring the activation of JNK, which phosphorylates IRS-1 in Ser residues and blocks the PI3K signaling pathway (Figure 3) (Özcan et al., 2004; 2006; Park et al., 2010; Zhou et al., 2012; Vazquez-Jimenez et al., 2016). Therefore, the proper regulation of SR Ca^2+^ levels is necessary to avoid the ignition of ER stress in the VSMCs. The RyRs, together with the SERCA pump, are key proteins involved in regulating the SR Ca^2+^ load. The diminished expression of the SERCA pump, together with the ER stress, are involved in the development of IR. In these settings, the altered PI3K/Akt/eNOS pathway in ECs will compromise NO production with the consequent decrease in PKG activation in the VSMCs, impairing vasorelaxation (Figure 3) (Madonna and Caterina, 2009; Vazquez-Jimenez et al., 2016). Similar alterations have been documented in experimental models of DM2. For instance, in the aorta of the diabetic (*db/db*) mouse, the expression of the SERCA2 pump was found decreased (Kimura et al., 2022); while in VSMCs of cerebral arteries, the time dependence of SR Ca^2+^ load recovery was impaired and the Ca^2+^ spark properties were diminished (Rueda et al., 2013). All these data suggest the presence of ER stress associated with a compromised SERCA pump function as common vascular alterations in prediabetes, MetS and DM2.

## 3 Role of intracellular Ca^2+^ signaling in VSMCs

Systemic blood pressure results from the force exerted by the heart on the wall of resistance arteries. The vascular tone of resistance arteries is determined by the level of contraction/relaxation of VSMCs surrounding the blood vessels, which relies on the efficient regulation of [Ca^2+^]_cyt_; therefore, changes in both Ca^2+^ influx and/or Ca^2+^ release from the intracellular Ca^2+^ stores of VSMCs directly regulate vasoconstriction/vasodilation of the blood vessels through the contractile machinery of the cells (Fernández-Velasco et al., 2014). While intracellular Ca^2+^ fluctuations are key to the contraction and relaxation of VSMCs, they also play a critical role in a vast array of other cellular functions. For instance, intracellular Ca^2+^ signals are involved in VSMCs’ proliferation and migration and are essential in vascular repair and remodeling after injury (Marchand et al., 2012). Intracellular Ca^2+^ signals also participate in cytoskeletal remodeling, promoting actin polymerization, and regulating the expression and function of integrins, altering VSMCs’ adhesion properties. PI3K, Ca^2+^-dependent protein kinases (CaMKs), Rho-activated protein kinases (ROCKs), and MAPKs are the main pathways in VSMCs migration (Gerthoffer, 2007).

Another mechanism by which neurotransmitters and hormones release Ca^2+^ from the internal Ca^2+^ stores involves the activation of G protein-coupled receptors (GPCRs), which activate phospholipase C, inducing the hydrolysis of phosphatidylinositol 4,5-bisphosphate to generate diacylglycerol and inositol 1,4,5-trisphosphate (IP_3_). The latter binds to the IP_3_ receptors (IP_3_Rs) embedded in the SR membrane. The opening of the IP_3_Rs produces global increases in [Ca^2+^]_cyt_ and the subsequent contraction of VSMCs.

It is also worth mentioning that to maintain Ca^2+^ homeostasis in VSMCs, the Store-Operated Ca^2+^ Entry (SOCE) is an important mechanism to replenish SR Ca^2+^ stores via increasing Ca^2+^ entry through the channel pore-forming protein Orai1. The SOCE mechanism is crucial for sustained contraction, vasculogenesis, vascular tone regulation, and VSMCs proliferation (Avila-Medina et al., 2018).

Therefore, a diverse set of Ca^2+^ handling proteins (for instance, channels, pumps, exchangers, signal transducers, buffers, etc.), collectively known as the Ca^2+^ toolkit, controls the wide range of Ca^2+^ signaling pathways in vascular tissues. All these are well-known aspects of intracellular Ca^2+^ handling in vascular cells and have been explained in depth in compelling reviews (Tykocki et al., 2017; Fan et al., 2019; Pereira Da Silva et al., 2022). This review centers on alterations in the expression or activity of the proteins that form the functional unit that participates in the regulation of the vascular tone (Ca_V_1.2, SERCA pump, RyRs, and BK_Ca_ channels) in experimental models with characteristics of prediabetes and MetS.

### 3.1 The functional unit that regulates the vascular tone and participates in vasorelaxation in VSMCs

As a multifunctional intracellular messenger, Ca^2+^ can signal both the contraction and relaxation of VSMCs; the fine-tuning of these signals is a crucial feature of smooth muscles. Particularly, elementary local Ca^2+^ signals produced by RyRs participate in vasorelaxation. Under physiological conditions, the local Ca^2+^ signals, also known as Ca^2+^ sparks, activate nearby BK_Ca_ channels that generate STOCs. STOCs have a key role in the control of arterial tone by shifting plasma membrane potential toward less positive values, which, in turn, limits Ca^2+^ influx through Ca_V_1.2 and diminishes [Ca^2+^]_cyt_ opposing vasoconstriction. Therefore, Ca^2+^ sparks and STOCs are a functional coupled unit that regulates arterial tone. The coordinated opening of RyR clusters produces the Ca^2+^ sparks, and their ignition is under the rapid and direct control of the Ca^2+^ content inside the SR Ca^2+^ stores (Essin et al., 2007). Cheranov and Jaggar (2002) demonstrated a positive relationship between the SR Ca^2+^ load and the Ca^2+^ spark frequency in VSMCs (Cheranov and Jaggar, 2002). To date, the most effective mechanism to trigger Ca^2+^ sparks in VSMCs appears to be the Ca^2+^ inside the SR, which is loaded primarily by the SERCA pump. Additional molecular mechanisms that control the frequency and spatio-temporal properties of Ca^2+^ sparks in VSMCs have also been shown; for instance, the removal of proteins such as phospholamban (PLN) (Wellman et al., 2001), RyR3 (Löhn et al., 2001), FK 506 Binding Protein of 12.6 kDa (FKBP12.6) (Ji et al., 2004), and sorcin (Rueda et al., 2006) increases the frequency of Ca^2+^ sparks in VSMCs.

Most recent evidence has further supported the participation of RyR2 in global and local SR Ca^2+^ release with the help of the first conditional knockout of RyR2 in VSMCs (SM-RyR2-KO mouse) (Kaßmann et al., 2019). Studies using the SM-RyR2-KO mouse showed that the RyR2 isoform plays a significant role in global caffeine-induced Ca^2+^ release within VSMCs, and that RyR2 expression is also a prerequisite for the formation of Ca^2+^ sparks, which limit arterial myogenic constriction to pressure (Kaßmann et al., 2019). Therefore, Ca_V_1.2, SERCA pump, RyRs, and BK_Ca_ channels form a functional unit that regulates the vascular tone; any alteration of these proteins would lead to changes in vasoconstriction or vasorelaxation. In the next section, we summarize and discuss the studies that report alterations in the expression or the activity of these proteins in experimental models with characteristics of prediabetes or MetS.

### 3.2 Alterations in the functional unit that regulate the vascular tone in experimental models with characteristics of prediabetes and MetS

In the settings of DM1 and DM2, vascular dysfunction has been extensively investigated and summarized in compelling reviews (Feener and King, 1997; Creager et al., 2003; Fernández-Velasco et al., 2014; Nieves-Cintrón et al., 2018; Li et al., 2023). For instance, when hyperglycemia is established in DM2, there is a downregulation in K_v_ and BK_Ca_ channel activity by mechanisms associated with oxidation, and an increase in Ca_V_1.2 activity via protein kinase A phosphorylation, hence disrupting Ca^2+^ signaling and resting membrane potential, leading to impaired myogenic tone and vascular reactivity (Nieves-Cintrón et al., 2021; Pereira Da Silva et al., 2022). Likewise, in the diabetic environment, the SERCA pump has been reported to be altered. For instance, in CA smooth muscle cells from diabetic dyslipidemic pigs, SERCA activity and protein expression were increased, which may induced SR Ca^2+^ overload (Hill et al., 2001). Conversely, a decrease in SERCA2 expression was found in the aorta of the diabetic (*db/db*) mouse without assessing its activity (Kimura et al., 2022); therefore, the data are still not conclusive. Also, in the diabetic (*db*/*db*) mouse, along with lower RyRs expression, a concomitant diminution of Ca^2+^ spark properties was reported in cerebral artery VSMCs, though Ca^2+^ spark frequency remained similar. Nevertheless, STOC frequency was depressed, perhaps due to a decrease in the BK channel β1/α subunit ratio found in *db/db* vascular tissues (Rueda et al., 2013). These results suggest that alterations in the functional unit that regulates vascular tone participate in diabetic vasculopathy.

Little information is available about the effects of prediabetes and MetS on this set of proteins (the Ca_V_1.2, the SERCA pump, the RyRs, and the BK_Ca_ channels) in the human vasculature. In this regard, an increased open probability of BK_Ca_ channels has been reported in human CA of atherosclerotic plaques with respect to BK_Ca_ channels from non-atherosclerotic CAs (Wiecha et al., 1997), which contrasts with the existence of BK_Ca_ channels with shorter open time and lower Ca^2+^ sensitivity in atherosclerotic human aorta, similar to the activity of BK_Ca_ channels in aorta with proliferative phenotype (Volotina et al., 1991). The lack of conclusive results may be related to the type of blood vessel studied (resistance vs cursive conductance artery).

In this review, we focus on the available information reporting changes in the expression and/or the activity of the above-mentioned proteins in experimental models with characteristics of prediabetes and MetS (Table 2).

**TABLE 2:** Alterations in the function and/or expression of Ca_V_1.2, SERCA pump, RyRs, and BK_Ca_ channels in vascular tissues of animal models with characteristics of prediabetes or MetS**.**

Experimental Model	Metabolic features	Treatment	Tissue	Protein	References
In swine					
Male and female young Ossabaw	↑ Body weight ↑ Total cholesterol ↔ Triglycerides ↔ Fasting glucose Impaired glucose tolerance	Atherogenic diet (11 months)	Coronary artery	↓ SERCA [FA] ↑ Ca_V_1.2 [FA]	Badin et al. (2018)
Male Ossabaw	↑ Total cholesterol ↑ LDL-C ↑ HDL-C Impaired glucose tolerance ↔ Triglycerides ↑ Insulin	Atherogenic HFD diet (43 weeks)	Coronary artery	↓ SERCA [FA]	Neeb et al. (2010)
Male Yucatan	↑ Total cholesterol ↑ LDL-C ↑ HDL-C ↔ Triglycerides	Atherogenic HFD diet (43 weeks)	Coronary artery	↓SERCA [FA]	Neeb et al. (2010)
Male Ossabaw	↑ Body weight ↑Total cholesterol ↔ Glycemia ↑ Triglycerides ↔ Insulin	Atherogenic HFD diet (6 months)	Coronary smooth muscle cells	↓ SERCA [FA]	Dineen et al. (2015)
Male Yucatan	↑ Total cholesterol ↑ LDL-C ↑ HDL-C ↔ Glycemia ↔ Triglycerides	Atherogenic HFD diet (8 weeks)	Coronary artery	↑SERCA [FA] ↑SERCA [WB]	Hill et al. (2003)
Male Yucatan	↔ Glycemia ↔ Body weight ↑ Total cholesterol ↔ Triglycerides	HFD diet (20 weeks)	Coronary artery	↔SERCA2b [WB]	Witczak et al. (2006)
In rat					
Male Obese Zucker	Mild hyperglycemia ↑ Triglycerides ↑ Cholesterol	---	Coronary artery	↑ BK_Ca_β1 [WB] ↔ BK_Ca_α [WB] ↑ Ca_V_1.2 [WB] ↓ RyR [FA] ↔ RyR [WB] ↔ SERCA [WB]	Climent et al. (2017), 2020
Male Obese Zucker	Impaired glucose tolerance ↑ Insulin ↑ Triglycerides ↑ Cholesterol Insulin resistance	---	Cerebral artery	↓ RyR [FA]	Katakam et al. (2014)
Male Obese Zucker	↑ Body weight Moderated hyperglycemia ↑ Insulin ↑ Cholesterol ↑ Triglycerides	---	Mesenteric artery	↑ Ca_V_1.2 [WB] ↑ RyR [WB] ↓ RyR [FA]	Sánchez et al. (2018)
Male JCR:LA-cp Rat	Obesity ↑ Triglycerides ↑ Insulin Impaired glucose tolerance	---	Aorta	↑ Ca_V_1.2 [FA]	Russell et al. (1994), Gupte et al. (2020)
Male Sprague-Dawley	Not determined	High Cholesterol diet (2%) for 30 weeks	Cerebral artery	↑ BK_Ca_β1 [WB, FA] ↔ BK_Ca_α [WB]	Bukiya et al. (2021)
Male Sprague-Dawley	↑ Insulin Dyslipidemia	Fructose-rich diet (66%) for 4 weeks	Cerebral artery	↔ BK_Ca_α [WB] ↓ BK_Ca_ [FA]	Erdös et al. (2002a), Erdös et al. (2002b), Erdös et al. (2004)
Male Sprague-Dawley	↑ Body weight ↑ Insulin ↑ Cholesterol ↑ Triglycerides ↔ Glycemia	High-fat and High-sucrose diet for 8 weeks	Thoracic aorta Mesenteric artery	↔ BK_Ca_α [RT] [WB] ↓ BK_Ca_β [RT] [WB] ↓ BK_Ca_ [FA]	Shangjian et al. (2011)
Male Sprague-Dawley	↑ Body weight ↑ Insulin ↑ Glycemia	HFD for 16–20 weeks	First-order cremaster muscle arterioles	↔ BK_Ca_α [RT] [WB] ↔BK_Ca_β1 [RT] ↓ BK_Ca_β1 [WB] ↔ BK_Ca_ [FA]	Howitt et al. (2011)
			Middle cerebral artery	↔ BK_Ca_α [RT] [WB] ↔BK_Ca_β1 [RT] ↑ BK_Ca_β1 [WB] ↔ BK_Ca_ [FA]	
Male Sprague-Dawley	↑ Insulin ↑ Triglycerides ↑ Glycemia	High fructose diet (60%)	Mesenteric lymphatic vessels	↓SERCA2a [IF]	Lee et al. (2020)

Arrows indicate decreased (↓), increased (↑), or no change (↔) in the expression of the proteins of interest, determined by immunofluorescence [IF], real-time qPCR [RT], Western blot, [WB], and/or functional activity [FA]. BK_Ca_ channels, large conductance Ca^2+^-activated K^+^ channels; Ca_V_1.2, L-type voltage-gated Ca^2+^ channel; HFD, high-fat diet; RyR, ryanodine receptors; SERCA pump, sarco/endoplasmic reticulum Ca^2+^ ATPase.

The SERCA pump is the most studied protein of the functional unit, it links IR and ER stress with the Ca^2+^ handling alterations. A dysfunctional SERCA was reported in CA smooth muscle cells of Ossabaw pigs fed with an atherogenic high-fat diet (HFD) for 6 months (Neeb et al., 2010; Dineen et al., 2015). In VSMCs of this animal model, the Endothelin-1-induced Ca^2+^ peak was decreased similarly to that of control cells in which the SERCA pump was inhibited by thapsigargin (Neeb et al., 2010; Dineen et al., 2015). Therefore, it was suggested that the SERCA pump contributes significantly to buffering the Endothelin-1 associated Ca^2+^ response, and its dysfunction impairs VSMC relaxation from Ossabaw pig CA (Neeb et al., 2010; Dineen et al., 2015). Similarly, after an atherogenic diet for 11 months, a MetS young swine model exhibited SR Ca^2+^ store dysregulation in CA, evidenced by the attenuated caffeine-induced SERCA activity (Badin et al., 2018). However, another study showed increased expression and activity of the SERCA pump in CA of male Yucatan pigs, as a compensatory mechanism (Hill et al., 2003), but SERCA2b protein expression levels was reported without changes in CA of male Yucatan swine subjected to a high-fat and high-cholesterol diet; though SERCA pump activity was not assessed (Witczak et al., 2006).

The OZR is also a relevant animal model for MetS in humans, which develops alterations in vascular reactivity. An increase in the expression of Ca_V_1.2 and the β1 subunit of BK_Ca_ channels has been consistently shown in CA from OZR, without changes in the expression of the BK_Ca_α subunit, nor in RyRs or the SERCA pump (Climent et al., 2017; Climent et al., 2020). While an increase in the expression of Ca_V_1.2 will favor more Ca^2+^ influx, the concomitant increase in BK_Ca_β1 subunit, which enhances the Ca^2+^ sensitivity of the BK_Ca_ channels, will counterbalance the otherwise abnormal Ca^2+^ influx, leading to a preserved basal tone despite obesity alterations (Climent et al., 2017; Climent et al., 2020). Another report also showed that cerebral arteries from Sprague Dawley rats fed with a high-cholesterol diet had increased protein levels of the BK_Ca_β1 subunit with augmented open probability of BK_Ca_ channels (Bukiya et al., 2021). Therefore, it was hypothesized that VSMCs would be hyperpolarized, which would decrease Ca^2+^ entry and contraction. However, cerebral arteries from OZ rats that exhibited IR and features of MetS, such as impaired glucose tolerance, hyperinsulinemia, hypertriglyceridemia, and hypercholesterolemia, showed a diminished generation of Ca^2+^ sparks and reduced vasodilation (Katakam et al., 2014). In addition, cerebral arteries from Sprague-Dawley rats fed with a fructose-rich diet for 4 weeks showed decreased functional activity of BK_Ca_ channels without changes in the BK_Ca_ protein expression (Erdös et al., 2002a; Erdös et al., 2002b; Erdös et al., 2004), which contrasts with no changes in BK_Ca_ activity but increased expression of BK_Ca_β1 subunit in cerebral arteries from Sprague-Dawley rats fed with HFD (Howitt et al., 2011). Moreover, a reduction in SERCA2a protein expression was demonstrated in mesenteric lymphatic vessels of Sprague-Dawley rats fed with HFD (Lee et al., 2020), suggesting that metabolic alterations impair SERCA expression or activity in other vessels besides arteries. Notably, the impairment of BK_Ca_ channel activity was associated with higher IR-induced oxidative stress because the loss of function of BK_Ca_ channels was prevented by scavenging ROS. Dysfunctional RyRs and BK_Ca_ channels would lead to a reduction in the control of vascular tone in the setting of fructose-induced IR in rats.

MetS features participate in altering the reactivity and wall mechanics of cerebral arteries in OZR (Brooks et al., 2015). Mesenteric arteries of adult OZR (8–10 weeks of age) that developed obesity, mild hyperglycemia, and hyperinsulinemia showed increased levels of RyRs, although with reduced activity. The expression of Ca_V_1.2 was also increased compared to lean Zucker Rats, and therefore, OZR showed augmented vasocontraction (Sánchez et al., 2018).

Alterations in the proteins of interest have also been reported in the JCR:LA-cp rat. This animal model was developed in 1978 with an autosomal recessive corpulent (cp) trait resulting from a premature stop codon in the extracellular domain of the leptin receptor (Diane et al., 2016). Homozygous JCR:LA-cp rats display the pathophysiology of obesity with a MetS-like phenotype. The aortas of JCR:LA-cp rats presented increased Ca_V_1.2 expression levels, which likely contributed to the increase in mean arterial blood pressure (Howitt et al., 2011). The consumption of high-sucrose during the pre-natal stage is also important for the physiological activity of VSMCs, because the offspring of rats that consumed high-sucrose during gestation showed decreased expression of the Ca_V_1.2, and the α and β subunits of the BK_Ca_ channel, together with the impairment of their functional activity (Feng et al., 2019).

Additional studies in experimental models have reported alterations in other important proteins for proper vascular function. For instance, the reduction in the bioavailability of vascular-derived NO, together with an increase in the systemic proinflammatory condition in the settings of MetS, augmented TNF-α levels inducing the abnormal remodeling of the resistance blood vessels, a condition that was alleviated by the acute treatment with the antioxidant tempol (Brooks et al., 2015). In the CA of the MetS Ossabaw pig model, it was reported a decreased expression and a loss of function of K_V_7 channels, which hyperpolarize VSMCs to induce relaxation (Chen et al., 2016). The reduced K_V_7 channel expression may lead to sustained histamine-induced contractions and reduced endothelium-dependent relaxation, both risk factors for coronary spasm (Chen et al., 2016).

It is also important to consider that the transition of VSMCs from the contractile phenotype in the healthy swine to the proliferative phenotype in mild atherosclerosis has been associated with increases in the SERCA activity, SR Ca^2+^ load, and the L-type Ca^2+^ channel function (Badin et al., 2022), and it deserves future studies. Interestingly, the dysregulation in the intracellular Ca^2+^ handling is similar in CA of Ossabaw miniature swine with respect to what has been found in coronary disease of humans (Badin et al., 2022), suggesting similar underlying mechanisms.

## 4 Discussion

Few studies have analyzed the effect of prediabetes and MetS on the functional unit that regulates vascular tone and vasorelaxation, and we provide evidence of a high variability in the outcomes. For instance, the expression and activity of the SERCA pump, RyRs, and BK_Ca_ channels increased, decreased, or remained unchanged depending on the experimental model (Table 2). These discrepancies may result from the broad diversity of animal models, species variability, and the duration, time, and type of diet to induce metabolic alterations. This variability complicates the identification of therapeutic targets and highlights the necessity to standardize the experimental models in prediabetes and MetS research. Furthermore, evaluating the proteins of the functional unit that regulates vasorelaxation is crucial for identifying new potential targets in vascular pathologies associated with prediabetes and MetS.

Moreover, in MetS, the vascular endothelium is also altered and can influence the VSMCs’ function. For instance, the ECs of mesenteric arteries from an experimental mouse model of MetS showed impaired ACh-induced vasorelaxation, while the vasorelaxation induced by SNP was enhanced, suggesting endothelial dysfunction (Aoqui et al., 2014). In another study with MetS patients, it was shown that the perivascular adipose tissue, which is in close contact with VSMCs and provides protection in healthy conditions, develops local inflammation, leading to the loss of its vascular protection effect (Greenstein et al., 2009).

**FIGURE 4:**
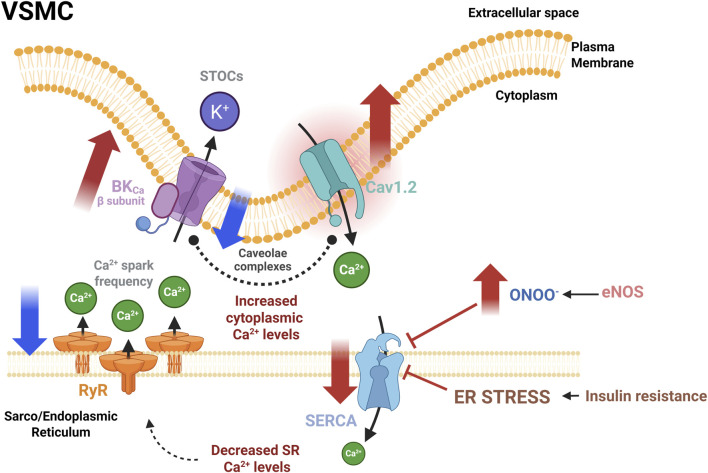
Dysregulation of the functional unit that controls the vascular tone in experimental models of prediabetes and MetS. Abnormal augmented activity of Cav1.2 is a consistent manifestation of vascular dysfunction in IR, a metabolic condition present in prediabetes and MetS. Also, a decrease in RyR activity, evidenced by decreased Ca^2+^ spark frequency, could be compensated by the BK_Ca_ β subunit overexpression. The Ca_V_1.2 increased expression seems to be a compensatory mechanism. Conversely, alterations in SERCA pump expression and activity are diverse. Conditions that promote an increase in peroxynitrite production may impair SERCA activity and induce ER stress, ultimately decreasing SR Ca^2+^ load and interfering with the vasorelaxation mechanism. Red solid arrows indicate changes in both the expression and the activity of indicated proteins; Blue solid arrows indicate changes in the activity only. BK_Ca_, large conductance Ca^2+^ activated K^+^ channels; Ca_V_1.2: L-type voltage-gated Ca^2+^ channel; eNOS, endothelial nitric oxide synthase; RyR, ryanodine receptor; SERCA, sarcoplasmic/endoplasmic reticulum Ca^2+−^ATPase; STOCs, Spontaneous transient outward currents. Created in BioRender.

Despite the discrepancies observed in the alterations reported in SERCA pump expression and function, we consider that both SERCA pump and Ca_V_1.2 are key participants in the vascular dysfunction in prediabetes and MetS by contributing directly to Ca^2+^ handling dysregulation (Figure 4). According to the literature, the excessive ROS production oxidizes the SERCA pump and consumes the NO generated by eNOS activity, forming peroxynitrite (ONOO-), an anion known for inhibiting the SERCA pump more severely in VSMCs than in ECs (Schmidt et al., 2004). The final effect is the disturbing SERCA pump functionality interfering with vasorelaxation (Rahate et al., 2020). Besides, under ER stress, the SERCA pump activity is also impaired. This condition perturbs the SR Ca^2+^ stores, reducing the Ca^2+^ spark frequency through the RyRs, altering the STOC frequency and, therefore, the vasorelaxation (Figure 4).

Diverse mechanisms attempt to compensate for the adverse effects of ER stress, like the overactivation of the key proteins involved in the intracellular Ca^2+^ handling, such as Ca_V_1.2, and the BK_Ca_β subunit, as a prevalent alteration in prediabetes and MetS (Table 2). In agreement with the reports analyzed in this review, the abnormally increased expression of Ca_V_1.2 would promote an augmented Ca^2+^ entry, enhancing vasoconstriction. This elevated Ca^2+^ influx may increase [Ca^2+^]_cyt_; however, in the presence of a dysfunctional SERCA pump, the loading of the SR Ca^2+^ stores is compromised, decreasing Ca^2+^ spark frequency and, therefore, impairing vasorelaxation. Another possible mechanism to compensate for these alterations is an augmented expression of the BK_Ca_β subunit, which favors BK_Ca_ channel activation. In fact, both Ca_V_1.2 and BK_Ca_ channels have been found in the caveolae, where they interact in the functional unit (Suzuki et al., 2013; D’Agostino et al., 2021). MetS is associated with lipid raft dysfunction through the imbalance of sphingolipid content, reduced plasma membrane fluidity by increasing saturated and decreasing polyunsaturated fats in lipid rafts, and cholesterol depletion in the plasma membrane, among some other alterations (Gianfrancesco et al., 2018; Liu et al., 2023). The lipid raft dysfunction impairs Caveolin-1 scaffolding in VSMCs. The lack of Caveolin-1 reduces the co-localization of Ca_V_1.2 and BK_Ca_ channels, impairing not only the direct coupling between these proteins but also the functional coupling between RyRs and BK_Ca_ channels, presumably increasing [Ca^2+^]_cyt_ and VSMCs contractility (Suzuki et al., 2013). However, the modifications in the subcellular distribution of these proteins have not been comprehensively evaluated in VSMCs, in the settings of prediabetes or MetS.

It is of importance to mention the lack of data about alterations in this functional unit in females with prediabetes or MetS. In this regard, the beneficial effects of 17-β-estradiol in women against abnormal vascular tone development, such as coronary arterial vasospasms (Hill and Muldrew, 2014), might also be used to recover the SERCA pump expression in females with prediabetes or MetS. This idea is supported by the data showing the upregulation of SERCA2b in pig coronary arteries via activation of the classic estrogen receptor pathway (Hill and Muldrew, 2014).

It is also worth mentioning the role of the Ca^2+^-sensing receptor (CaSR), a GPCR expressed in the vasculature, which plays a crucial role in regulating intracellular Ca^2+^ homeostasis by activating phospholipase C-dependent pathways (Guo et al., 2018). Particularly in VSMCs, CaSR contributes to blood pressure regulation and vascular tone by influencing smooth muscle contraction. Hence, CaSR has been associated with several pathological conditions, such as DM2. Studies have shown that the resistance arteries of diabetic rats exhibit lower levels of CaSR expression, which may disrupt Ca^2+^ homeostasis and contribute to the development of hypertension and other vascular dysfunctions associated with DM2 (Weston et al., 2008). However, the implications of CaSR in the vasculature of prediabetic or MetS patients remain unexplored.

## 5 Therapeutic approaches

The current therapies help improve the clinical signs of MetS, but it would be beneficial to address attention to the Ca_V_1.2, the SERCA pump, the RyRs, and the BK_Ca_ channels as pharmacological targets. Researchers have also assessed antidiabetic drugs for MetS-associated complications. For instance, liraglutide, a glucagon-like peptide 1 (GLP-1) receptor agonist, is commonly used as an antihyperglycemic drug in DM2 patients (Grannes et al., 2024). Besides its effects on glucose metabolism, liraglutide has shown its efficacy in ameliorating insulin sensitivity in obese and prediabetic patients (Mashayekhi et al., 2024). Apparently, liraglutide acts as a weight-loss inducer by reducing the soluble CD163, a macrophage proinflammatory marker associated with obesity, MetS, and IR before DM2 establishment (van der Zalm et al., 2020; Grannes et al., 2024). Besides the action of liraglutide at the systemic level, this drug improves the function of the vascular endothelium and reduces oxidative stress in MetS patients when combined with metformin (Liu, 2024). Specifically, in ECs from DM2 patients, liraglutide decreases JNK activation, ameliorating ER stress by improving eNOS activity (Bretón-Romero et al., 2018). The acute effect of another GLP-1 receptor agonist, exenatide, has also been tested on the intracellular Ca^2+^ regulation in VSMCs from the carotid artery of Ossabaw swine with MetS. However, the authors found that exenatide did not affect Ca^2+^ regulation or SERCA activity in VSMCs of the MetS swine (Dineen et al., 2015). Since the onset of ER stress is already occurring in MetS, it would be remarkably interesting to study the effect of these drugs on the prediabetic condition. Additional therapeutic approaches include antidiabetic agents such as gemigliptin and canagliflozin, and extra virgin olive oil phenols which promote vasodilation by a mechanism involving the activation of K_V_ channels and the SERCA pump in aortic smooth muscle cells and mesenteric resistance arteries (Jung et al., 2020; D’Agostino et al., 2021; Heo et al., 2021), although its effects on the vasculature of prediabetic and MetS patients are not yet explored.

Another possible therapeutic approach for palliating vascular dysfunction in MetS patients involves targeting UPR-related ER stress. In this regard, chaperone-like tauroursodeoxycholate and 4-phenyl butyrate are used because they fold and stabilize ER proteins in several cell lines (Mohan et al., 2019). Notably, chaperone-like tauroursodeoxycholate effectively reduces aortic clot formation and improves atherosclerotic lesions and ER stress in CVD, while improving insulin sensitivity and reducing inflammation in obese patients (Kars et al., 2010; Bouchecareilh et al., 2011). Also, CDN1163, a small SERCA pump activator, restores Ca^2+^ homeostasis and improves glucose tolerance in *ob*/*ob* mice, attenuating metabolic disorders (Kang et al., 2016). Finally, the use of chemical chaperones may also have the potential to prevent amyloid formation in the ER. For instance, Tafamidis, a drug that prevents extracellular amyloidogenesis, has shown benefits in reducing cardiovascular mortality (Marciniak et al., 2022). Therefore, future attention should be given to studying the effects of CDN1163 and Tafamidis on the Ca^2+^ dynamics of VSMCs.

As previously mentioned, oxidative stress is another major component of vascular dysfunction in MetS and prediabetes. The deleterious effect of excessive ROS on SERCA pump activity must be recognized. ROS induces the irreversible oxidation of the SERCA pump, thus inhibiting NO-mediated SERCA activation. Also, ROS provokes NO overconsumption in prediabetes, preventing glutathionylation-mediated SERCA activation (Tong et al., 2010). Thus, the use of phytochemical extracts with antioxidant potential should be explored as a promising area of study in the prevention and treatment of vascular dysfunction in prediabetes and MetS. Isothiocyanates extracted from cruciferous vegetables, such as sulforaphane and its precursor glucuronide, have been used to prevent and control DM2, cardiometabolic syndrome, IR, and obesity (Wang et al., 2022). Hence, besides the pharmacological strategies already explained, it is crucial to regulate oxidative stress levels to maintain proper SERCA pump activity and, thus, Ca^2+^ homeostasis in blood vessels.

Several reports suggest sulforaphane upregulates the expression of the transcription factor Nrf2 (nuclear factor erythroid 2-related factor 2), and as a result, increases the activity of several proteins involved in the metabolism of glucose (IRS-1, PDK1, Akt), lipids (PPAR, adiponectin), oxidative stress (SOD, CAT, GSH) and inflammatory signaling (TNF-α, JNK, ERK) (Wang et al., 2022); resulting in the enhancement of lipid metabolism, glucose transport, and control of oxidative stress levels. Although the effects of sulforaphane have been widely studied on DM2 models, some reports suggest its contribution to improving metabolic markers associated with IR in prediabetes (Zandani et al., 2021); however, its potential role in treating prediabetic vascular dysfunctions remains to be explored. Also, Chrysin, a flavonoid present in honey and several plants, exhibits pharmacological properties on Nrf2. Chrysin counteracts ER stress by inhibiting the PERK signaling in the myocardium of animals with a HFD, improving the lipid profile (Yuvaraj et al., 2024). Concerning vascular tissues, Chrysin inhibits endothelial inflammation and contributes to vasorelaxation mechanisms and the regulation of intracellular Ca^2+^ levels by reducing the Ca_V_1.2-mediated Ca^2+^ influx and inhibiting the IP_3_R-mediated SR Ca^2+^ release (Tew et al., 2023). Nevertheless, researchers have not yet explored the effects of these phytochemicals on prediabetic models.

A novel option to improve the vascular function in prediabetes and MetS should consider the recovery of the SERCA pump activity by the phosphorylation of PLN. For instance, the activation of the adenosine 5‘-monophosphate (AMP)-activated protein kinase (AMPK) is beneficial for the vasomotor function of resistance arteries by increasing PLN phosphorylation at Thr17; thus, activating SERCA could be favorable in MetS models (Schneider et al., 2015). Moreover, the sodium-glucose cotransporter 2 inhibitors empagliflozin and canagliflozin have been shown to activate AMPK by inhibiting the mitochondrial respiratory complex I (mechanism proposed for canagliflozin). This inhibition increases the AMP/ADP ratio promoting the phosphorylation of the AMPK α-subunit at Thr172, which activates AMPK (Hawley et al., 2016). In its non-phosphorylated state, PLN inhibits SERCA pump activity; however, phosphorylation at Thr17 diminishes this inhibitory effect, leading to increased SERCA activity. This upregulation of the SERCA pump increases SR Ca^2+^ uptake, improving vasomotor functions in resistance arteries. Additionally, empagliflozin has been shown to prevent diabetes-induced reduction in eNOS phosphorylation in myocardial tissue of diabetic mice and partially restored the endothelium-dependent vasorelaxation in the thoracic aorta (Zhou et al., 2018). Therefore, the therapeutic use of the sodium-glucose cotransporter 2 inhibitors might be beneficial for the vascular function in prediabetes and MetS.

## 6 Conclusion

Although prediabetes and MetS are distinct clinical conditions, both constitute latent risks for the development of DM2, and both share hyperinsulinemia and IR as key pathophysiological characteristics. Among the different tissues affected by these metabolic alterations, VSMCs are impacted, contributing to vascular dysfunction and an increased cardiovascular risk.

In prediabetes and MetS, vascular dysfunction induced by IR includes VSMCs proliferation, vasoconstriction, and proinflammatory activity, also activating molecular pathways associated with ER stress. In turn, ER stress plays a critical role in Ca^2+^ handling in VSMCs, implying that hyperinsulinemia and IR indirectly alter the expression and function of the proteins of the functional unit (Ca_V_1.2, SERCA, RyRs, and BK_Ca_), dysregulating intracellular Ca^2+^ handling and impairing vascular tone. A consistent finding across the experimental models is the increased activity and/or expression of Ca_V_1.2, suggesting that this channel could be a potential therapeutic target because the undesired gain of function favors abnormal VSMC contraction. Conversely, the impact of these pathologies on the SERCA pump remains unclear. Therefore, evaluating changes in the expression or function of the SERCA pump is essential to understanding how vascular relaxation is impaired in experimental models of prediabetes and MetS. In this sense, studies have reported that either overexpression or the allosteric activation of the SERCA pump improves intracellular Ca^2+^ homeostasis in VSMCs of experimental metabolic models (Ferrandi et al., 2013).

Overall, a deeper understanding of the underlying molecular mechanisms in prediabetes and MetS that participate in altering the expression or the activity of the Ca_V_1.2, the SERCA pump, the RyRs, and the BK_Ca_ channels in the vasculature, will help to identify early events in diabetic vasculopathy, and to design prompt therapeutical interventions to stop the progression of the disease.

## Abbreviations

AMPK, adenosine 5′-monophosphate (AMP)-activated protein kinase; BIP/GPR78, binding immunoglobulin protein/glucose-regulated protein 78; BKCa, large-conductance Ca^2+^-activated K+ channels; CA, coronary artery; eNOS, endothelial nitric oxide synthase; ECs, endothelial cells; Ins, insulin; InsR, insulin receptor; IR, insulin resistance; IRS, insulin receptor substrate; JNK, c-Jun NH2-terminal kinase; MetS, Metabolic Syndrome; NO, nitric oxide; PI3K, Phosphatidylinositol 3-kinase; ROS, reactive oxygen species; RyR, ryanodine receptor; SERCA pump, sarco/endoplasmic reticulum Ca^2+^ ATPase; STOCs, spontaneous transient outward currents; VSMCs, vascular smooth muscle cells.
